# Log-binomial models: exploring failed convergence

**DOI:** 10.1186/1742-7622-10-14

**Published:** 2013-12-13

**Authors:** Tyler Williamson, Misha Eliasziw, Gordon Hilton Fick

**Affiliations:** 1 Departments of Family Medicine and Public Health Sciences, Queen’s University, Kingston, ON, Canada; 2 Department of Public Health and Community Medicine, Tufts University, Boston, MA, USA; 3 Department of Community Health Sciences, University of Calgary, Calgary, AB, Canada

**Keywords:** Log-binomial, Non-convergence, Failed convergence, Relative risk, Method of maximum likelihood, Log relative risk, Likelihood estimation, Maximum likelihood estimates, Logistic regression alternatives

## Abstract

**Background:**

Relative risk is a summary metric that is commonly used in epidemiological investigations. Increasingly, epidemiologists are using log-binomial models to study the impact of a set of predictor variables on a single binary outcome, as they naturally offer relative risks. However, standard statistical software may report failed convergence when attempting to fit log-binomial models in certain settings. The methods that have been proposed in the literature for dealing with failed convergence use approximate solutions to avoid the issue. This research looks directly at the log-likelihood function for the simplest log-binomial model where failed convergence has been observed, a model with a single linear predictor with three levels. The possible causes of failed convergence are explored and potential solutions are presented for some cases.

**Results:**

Among the principal causes is a failure of the fitting algorithm to converge despite the log-likelihood function having a single finite maximum. Despite these limitations, log-binomial models are a viable option for epidemiologists wishing to describe the relationship between a set of predictors and a binary outcome where relative risk is the desired summary measure.

**Conclusions:**

Epidemiologists are encouraged to continue to use log-binomial models and advocate for improvements to the fitting algorithms to promote the widespread use of log-binomial models.

## Introduction

One of the most basic epidemiological tenets is risk. It is intuitive and easily understood and explained to a wide audience. It is the conditional probability of an individual having the outcome of interest given a particular set of risk factors. Usually, it is of interest to frame risk as a comparison between two groups and one method for summarizing this comparison is the relative risk (RR) or the risk ratio. The relative risk, in its simplest form, is the ratio of two conditional probabilities,

$$\text{RR}=\frac{p_{1}}{p_{0}}$$

where *p*_1_ is the probability of the outcome for those exposured and *p*_0_ is the probability of the outcome for those unexposed. The simplicity of this definition makes it easily conveyed to a wide audience that may include clinicians, policy makers, or the general public. More generally, this ratio can be framed to reflect the presence and absence of an exposure either as an assumed common RR, after consideration of potential confounders, or as a set of stratum specific RRs after consideration of modifiers.

Yet, in spite of this, odds ratios (ORs) rather than RRs are the most frequently reported summary metric for reporting binary outcomes in modern epidemiological investigations [1]. The odds ratio, is a ratio of two conditional odds,

$$\text{OR}=\frac{p_{1}/(1-p_{1})}{p_{0}/(1-p_{0})}$$

where *p*_1_ and *p*_0_ are defined as above. ORs are frequently reported in a variety of settings. In case-control studies, ORs remain definitive [2]. But ORs are also reported in settings where most epidemiologists would regard the RR as the preferred measure of association [1]. In response to criticism of this practice, some would cite the well known fact that probability and odds are very close when the probability is itself small, the so-called rare-disease assumption [3]. However, another reason that ORs are reported in inappropriate settings is the current perception that there is not a viable alternative to logistic regression (which provides ORs) for modelling risk, particularly one that offers RRs rather than ORs.

The majority of work to-date on log-binomial models has been focused on trying to find solutions to the observed problem of failed convergence. Some of that work has provided reasonable approximations to the RR. However, unlike other papers on the subject, this work explores some possible reasons for failed convergence and provides potential solutions without resorting to an approximate solution.

### Generalized linear models

Modelling ORs is done through the use of logistic regression, a type of generalized linear model that uses the logistic function to link a dichotomous outcome (assumed to follow a Bernouilli distribution) to a set of explanatory variables (called the linear predictor when the variables are included in a linear way).

(1) $$\log\left(\frac{p}{1-p}\right)=\overset{j}{\underset{i=0}{\sum }}\beta_{i}x_{i}$$

A log-binomial model is a cousin to the logistic model. Everything is common between the two models except for the link function. Log-binomial models use a log link function, rather than a logit link, to connect the dichotomous outcome to the linear predictor.

(2) $$\log p=\overset{j}{\underset{i=0}{\sum }}\beta_{i}x_{i}$$

One immediate consequence of this change is the interpretation of the coefficients. In equation 1 the *β*_*i*_’s refer to differences in the log odds while in equation 2 the *β*_*i*_’s refer to differences in log risks. Except in some very special cases, there are no easy ways to link the coefficients from a logistic regression to those in a log-binomial unless one references the rare-disease assumption mentioned above.

If the intention is to report relative risks, then a log-binomial model allows easy access to an estimate of the relative risks, compared to logistic regression. However, this perceived gain comes at a cost. Both the logistic and log-binomial models are attempting to describe the relationship between a set of explanatory variables and the probability of a specific outcome. Probabilities are strictly defined between zero and one. The logit link maps the probability of the individual having the disease to the entire real line. The log-link function maps the probability of disease onto the negative real line, requiring the constraint that a linear predictor must be negative. This must hold true for all viable combinations of the explanatory variables to ensure that the implied probability is between zero and one. This simple constraint is one of the costs of choosing to model relative risk and is implicated in the estimation challenges for log-binomial models. That is, for log-binomial models, the parameter space for the set of regression coefficients is bounded, introducing the opportunity for estimation challenges.

The boundedness of the parameter space means that the likelihood function, the function that is maximized to estimate the model parameters, is only defined within that parameter space. Further, trying to maximize these likelihood functions acknowledging these boundaries is frequently problematic when using standard methodologies. The next section outlines some of the most popular methods that have been developed to deal with these problems.

Recently there was a paper published in Stroke [4], where in the statistical methods section the authors indicated that: “As a first approach to the multivariable analysis, we used a log-binomial model, but owing to the sparseness of data, this failed to converge. Therefore, we opted for a Poisson regression with robust variance estimator according to the SAS GENMOD procedure [5].” This type of statement is becoming increasingly common in top-tier medical journals. Researchers are recognizing the value of employing log-binomial models to represent their data. However, in the face of failed convergence, feel compelled to adopt one of the many workarounds, or resort to logistic regression, to even obtain any estimates at all. However, we submit that there may be circumstances where researchers may not have to abandon their log-binomial model, as a proper solution may be accessible.

### Existing workaround methods

Several papers have been published summarizing the methods currently available for the “approximate modelling of RRs” [6,7]. These papers all characterize the merits and demerits of the workarounds that have been suggested. The emphasis of this article is not to detail all of these methods; however, it is worth noting that, to date, almost all research on log-binomial models can be circumscribed to this category.

Wacholder was one of the first to articulate the estimation challenges inherent in estimating log-binomial models and was one of the first to propose a work around [8]. His suggestion was to evaluate the current fitted values at a given stage in the likelihood maximizing process [after each iteration in the search] and if any fitted values were outside the boundary space to set the fitted values to values known to be inside the space. A few years later, Lee and Chia [9] advocated that Cox regression could be adapted to approximate the solution if one built a dataset where every person had a pre-set and fixed follow-up time. Schouten [10] proposed the duplication of each case with the outcome of interest and suggested that modelling the log of the odds for the modified data might be the same as the log-binomial model for the unmodified data. Zhang and Yu [11] make use of a well-known method for converting odds ratios to relative risks using a baseline prevalence and then encouraged the use of logistic regression followed by the conversion of the OR to a RR using that conversion method. Another method has come to be called the COPY method [12]. With the COPY method, a large number of copies of the original dataset are appended to the original single copy of the data. Then, for one of the copies of the dataset, the outcome is switched for every observation in that copy and the model is then fit to the enlarged dataset with the necessary adjustments to the standard errors.

Yet another method for approximating the solution is the modified Poisson method proposed by Zou [13]. The modified Poisson regression method has gained the most attention in the literature and is growing in use. Advocates of the method suggest that the key advantage is that the failed convergence issues are practically non-existent [14]. This is due, in part, to the fact that Poisson regression is concerned with the log of expected counts and not the log of probabilities. Per se, there is no requirement that the linear predictor be constrained to be negative with a Poisson regression. Consequently, it is common that some positive fitted values are offered by the modified Poisson approach. Some authors have suggested that these can safely be ignored and that this should only be the case when the estimate is near a boundary [14]. However, presumably, the near boundary cases are some of the circumstances where one might expect failed convergence from a log-binomial model, so using a Poisson model here is likely to give probabilities outside the allowable space. While this method seemingly resolves the convergence issues, we cannot be satisfied with a method that gives fitted probabilities that are larger than one.

As previously mentioned, others have published work comparing the existing methods for approximating log-binomial models. This work takes a different approach to the problem. That is, that the problem is not the model itself but rather the limitations of the estimating algorithms to properly maximize the likelihood function. We submit that failed convergence does not imply that the model is inestimable. In fact, with a careful examination of the problem many non-convergent log-binomial models can be estimated after a simple reparametrization of the model or by using a different maximization technique, or perhaps the solution may be as simple as using a different software package.

## Analysis

### Simple models and failed convergence

To understand the mechanism of failed convergence, the simplest possible scenarios where failed convergence could occur were sought. The simplest of all log-binomial models is the model with a single binary predictor, as it effectively reproduces a 2×2 table. It is not surprising or interesting to observe failed convergence when there are zero cells in the 2×2 table as failed convergence could reasonably be expected from logistic regression for the same data. Therefore, every unique 2×2 table, with non-zero cells, for the fixed sample sizes of *n* = 20,25,…,70, were fit using a log-binomial model with a single binary predictor in both R (version 2.12.1) [15] and STATA (version 11.1) [16] and not a single case of failed convergence was observed. The next simplest model would be one with a single predictor that takes on three levels. Specifically, we explored models with a predictor, *X*, which was assumed to be linearly related to the log of the probability of the outcome and had only three possible values, *X* = -1,0,1.

(3) $$\log(p)=\beta_{0}+\beta_{1}X$$

The data could then be summarized using a single 3 × 2 table. Again, every possible 3 × 2 table, for samples of size *n* = 20,25,…,60, was fit using the log-binomial model in equation 3 in R [15] and STATA [16]. In total, more that 7.6 million unique 3×2 tables were examined. In R [15], approximately 3% (≈ 225 000) of these tables failed to converge after 100 iterations. It is from these 3×2 tables that the examples used below are drawn. Certainly, there are countless examples that could be chosen of the non-convergent log-binomial models, many of which have been published in top-tier medical journals. However, by choosing to use the simplest log-binomial models that demonstrate the point, two advantages are gained. First, using a model with only two parameters, allows the visualization of the log-likelihood function and the relevant parameter space. Second, the issues of non-convergence are not masked by the complexity of the model. The intention of what is presented next is an exploration of the fundamental concepts underpinning the estimation of log-binomial models. This can be adequately demonstrated with simple models and extensions to the more general setting follow naturally.

It is also important to mention that the majority of this work was done in R and STATA; however, failed convergence is not a problem isolated to these two software packages. Failed convergence was also observed in SAS (version 9.2) [17] and SPSS (version 19) [18] for various datasets. Software specific differences are discussed in Appendix 1 - Statistical software.

### Failed convergence

In general, generalized linear models are fit by maximizing the log-likelihood function, where the resultant maximum is referred to as the maximum likelihood estimate (MLE). Failed convergence occurs whenever the maximizing process fails to find the MLE. Further, estimation challenges can be grouped based on the location of the true maximum of the log-likelihood function, relative to the parameter space. Specifically, the maximum of the function can reside in one of three different locations: on the boundary of the parameter space (i.e., where the linear predictor equals 0); in the limit (i.e., as the linear predictor heads towards -*∞*); or inside the parameter space. These three regions span the entire parameter space and are mutually exclusive. Below are three sections that examine each of these scenarios individually including possible causes for the observed failed convergence and potential solutions if one can identify which of the three scenarios they are encountering.

#### *Maximum on a finite boundary*

It is not surprising that if the log-likelihood function is maximized on the boundary of the parameter space then an iterative method may have problems finding it as the algorithm may inadvertently step into an illegal space. Therefore, boundary issues are often assumed to be at fault when observing failed convergence with a log-binomial model. While this is the case occasionally, this should not be regarded as the only cause of failed convergence. Further, if one can positively identify the situations where the true maximum does lie on the boundary of the parameter space then the search for the maximum can be restricted to the boundary and, through a simple reparametrization of the model, a solution may frequently be found. Consider Table 1.

**Table 1:** Example dataset where the log-likelihood is maximized on the boundary of the parameter space

	**(X = -1)**	**(X = 0)**	**(X = 1)**	
(Y = 1) Disease	10	18	5	33
(Y = 0) No Disease	8	9	0	17
	18	27	5	50

Although the data is relatively simple, when model 3 is fit to this data using R [15], STATA [16], and SPSS [18] the model fails to converge. Perhaps not all that surprising given that for all subjects with *X*=1 only the outcome of interest was observed. Interestingly, when this data is fit using SAS [17] the algorithm converges to the proper solution but reports that the convergence is questionable given that it appears to be on the boundary.

**Figure 1:**
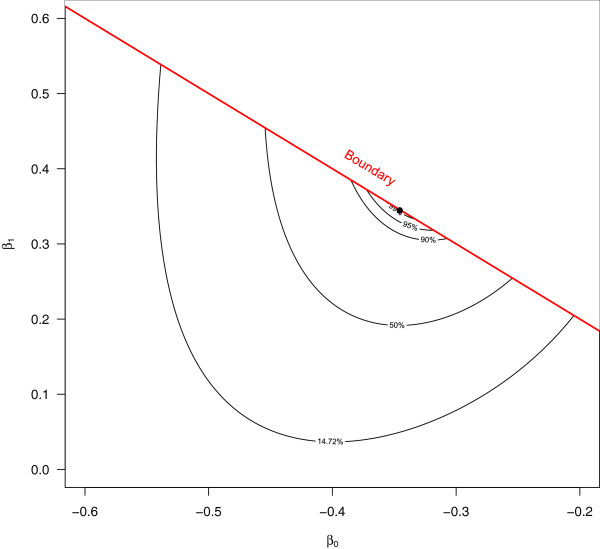
**Log-relative likelihood contours for a log-binomial model with data in Table**1**.**

To visualize the problem, the contours of the log-relative likelihood function are given in Figure 1. When viewing the log-likelihood function in this way, the observer can make meaningful statements about the shape of the function. For example, values inside the 14.7% relative likelihood region correspond approximately to the familiar 95% confidence interval [19]. The choice of 50%, 95% and 99% relative likelihood levels is somewhat arbitrary but, nevertheless, provide the relative plausibility of the parameter estimates inscribed by their respective regions. Estimates inside the 50% relative likelihood region are at least half as plausible as the MLE, while values inside the 95% and 99% relative likelihood regions are nearly and very nearly as plausible as the MLE. Additionally, the parameter space boundary is also indicated on the figure.

It is clear from Figure 1 that the log-likelihood is maximized on the boundary of the parameter space. Accordingly, it is not surprising that many software packages fail to locate the maximum as the iterative methods used in fitting the model may inadvertently iterate to an illegal place and cause the algorithm to fail. Curiously, the SAS algorithm iterates to the MLE. However, as discussed in Appendix 1 - Statistical software, while SAS finds this example correctly, there are other examples where it fails.

Nevertheless, if an analyst can properly identify situations where the solution is on the boundary, as is the case in the example, another, more reliable solution, can be employed. A simple reparameterization of the model makes the MLE readily available to all the software packages. For this particular dataset, the boundary of interest is the set of all points for which *β*_0_+*β*_1_ = 0, or equivalently *β*_0_ = -*β*_1_. Therefore, along this boundary the model can be rewritten as

(4) $$\begin{array}{l} \log(p)=\beta_{0}+\beta_{1}X_{1}\\ \;=-\beta_{1}+\beta_{1}X_{1}\\ \;=\beta_{1}(X_{1}-1)\\ \;=\;\beta_{1}V_{1}, \end{array}$$

where *V*_1_ is a new variable defined as (*X*_1_-1). If this model is fit, where the constant term is excluded and the single predictor is *V*_1_, then the model converges quickly to provide an estimate of the MLE using standard statistical software. The reparameterization has incorporated the knowledge that the solution resides on the boundary and the estimation becomes routine.

#### *Maximum in the limit*

In contrast to the situations where the estimate is on the finite boundary are the situations where the maximum is attained in the limit. These types of estimation problems are not, however, unique to log-binomial models. It is not uncommon for a logistic regression model to report problems if the data are such that for a particular subgroup only the outcome of interest is observed or if perhaps no outcomes are observed. With respect to log-binomial models, this usually occurs when the model attempts to estimate a risk of zero. For example, one could consider the situation where there were no observations for *Y* = 1, such as the data given by Table 2. Observing failed convergence of any model in this circumstance would not be noteworthy and, as expected, the same is true for the log-binomial model. Essentially, the log-likelihood function is increasing asymptotically towards zero as *β*_0_→-*∞*. The flat region this creates is problematic for the iterative fitting algorithm and the process fails.

**Table 2:** Example dataset where the log-likelihood function is maximized in the limit

	**(X = -1)**	**(X = 0)**	**(X = 1)**	
(Y = 1) Disease	0	0	0	0
(Y = 0) No Disease	17	21	12	50
	17	21	12	50

#### *Maximum inside the parameter space*

Observing failed convergence in the limiting or boundary cases is, in a sense, predictable. However, if the solution resides inside the parameter space (i.e., not on a boundary or in the limit) then observing failed convergence, when a finite maximum exists, should be properly regarded as a failure of the numerical method. Consider the data presented in Table 3.

**Table 3:** Example dataset where the log-likelihood is maximized inside the parameter space

	**(X = -1)**	**(X = 0)**	**(X = 1)**	
(Y = 1) Disease	2	14	2	18
(Y = 0) No Disease	2	3	17	22
	4	17	19	40

**Figure 2:**
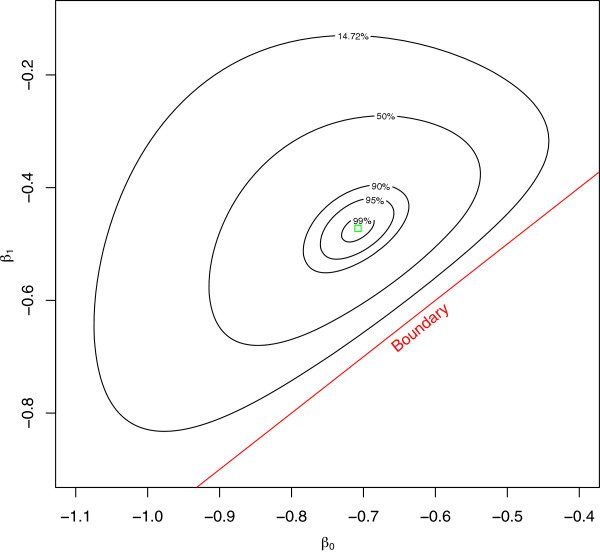
**Log-relative likelihood contours for a log-binomial model with data in Table**3**.**

Fitting a log-binomial model to this data ends in failed convergence in R [15], STATA [16], and SPSS [18] after 100 iterations. Yet, SAS [17] manages to report convergence after only a few steps. Also, the corresponding logistic regression model routinely converges in all four software packages. Naively, one might assume that the solution resides on a boundary given that the logistic regression models were so easily estimable; however, looking at the log-relative likelihood contours given in Figure 2, this is clearly not the case.

It can be shown that this function is unimodal and concave down in the region near the MLE, yet for some reason this model fails to converge in three of the mainstream statistical packages. Also, as previously mentioned, this type of data is not a peculiar dataset. In the simulation work described above, more than 200 000 similar datasets were found that, when fit using the same log-binomial model, would cause one or all of the software packages to fail to converge in spite of having a finite maximum inside the allowable space. Further, while these datasets came from relatively small samples, all of them could be considered plausible data coming from real-world settings. Readers that are interested in the technical details of this example are directed to Appendix 2 - Technical appendix where the log-likelihood, score and Hessian are explicitly provided. A more complete detailing of the general form of the log-likelihood function for all log-binomial models is outside the scope of this manuscript.

Certainly, the issues of failed convergence are software dependent and a more complete detailing of the software specific differences is included in Appendix 1 - Statistical software. As previously mentioned, in SAS this model converges rather routinely. However, there are other circumstances where SAS may converge to a place outside the parameter space (see Appendix 1 - Statistical software).

**Figure 3:**
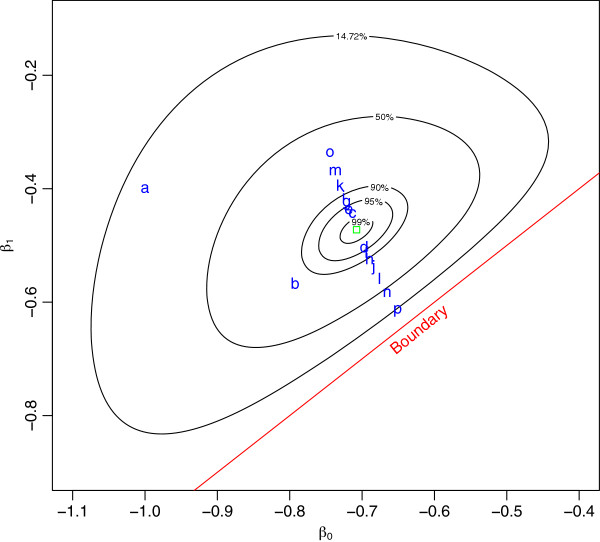
**Log-relative likelihood contours for a log-binomial model with data in Table **3**, with iteration steps.**

Figure 2 clearly demonstrates that the log-likelihood function for this model is unimodal and concave down. Presumably, any numerical optimization algorithm ought to be able to find the maximum of a function that is strictly decreasing away from the maximum. However, in R, as well as in STATA and SPSS, the fitting process iterates through hundreds of steps without declaring convergence. Looking closely at the fitting process for R demonstrates the problem. Oddly, the log-binomial fitting process has the iterations move quickly towards the MLE but then progressively move farther and farther from the MLE (see Figure 3). Even when starting values inside the 14.7% relative likelihood region are supplied, which are analogous to values drawn from within the respective 95% confidence intervals, the problem persists.

The reason that the model fails to report convergence is that the estimating algorithm enters what appears to be an infinite loop. The iterative process moves progressively further and further from the MLE. Eventually the next step would be to an illegal place and the algorithm self-corrects to land quite close to the MLE. Then resumes moving progressively away from the MLE again. In this case, it is an iteration loop of 22 steps, with each iteration moving further away from the MLE until the 22nd where the estimate is quite close to the MLE, but not the MLE. This phenomenon of going beyond the MLE is known as overshoot and, according to Lange [20], is usually ascribed to “the Hessian not being *well behaved* in the neighbourhood of the desired root”. This peculiar behaviour has been observed when trying to fit other log-binomial models or when using different data. Yet, the contours clearly show the existence of a unique finite maximum inside the parameter space.

### Brute force maximization

This work has shown that failed convergence may occur in very simple settings. However, in all of these scenarios, with the exception of the case where the MLE is achieved in the limit, the MLE could be approximated from the log-relative likelihood contours. In many of these cases, declaring these models “non-convergent” and abandoning their estimation is premature, provided that an alternative estimation approach can be found.

The increase in desktop computing power provides another option for dealing with observed failed convergence besides abandoning the model altogether. That is, brute force maximization [21,22]. When all else fails, a rudimentary approach can be taken to estimating the maximum of a log-likelihood function. Revisiting the data given in Table 2, applying a brute force maximization approach offers the MLE quickly and easily. In a way similar to what is done to generate a contour plot, a simple grid of defined precision is placed over a region of the parameter space and the log-likelihood function calculated at each intersection on the grid. The maximum of those points was then found and the process repeated with a smaller, more precise, grid centred at the current estimate of the maximum. This process can be repeated several times until a predefined precision is obtained. While this method is rudimentary and somewhat inefficient, the ability to rescue many log-binomial models that seemed otherwise inestimable may be of great value for the researcher who has invested perhaps years into collecting the data only to find that the desired log-binomial model fails to converge.

Undoubtedly, there are obvious criticisms of this type of approach including the fact that standard errors are not included as part of the estimating process and using the point estimates alone is useless. Nevertheless, having an estimate of the MLE can easily lead to subsequent estimation of the Fisher Information matrix and consequently estimation of the appropriate standard errors. Alternatively, the 14.7% relative likelihood region could also readily be used for calculating approximate 95% confidence intervals for log-binomial model parameters as suggested by Kalbleisch [19]. Relative likelihood intervals have the advantage of being asymmetric and are bound within the parameter space, whereas standard Wald-type intervals would not be asymmetric and could easily include values outside the parameter space.

However, in the short term, one can simply provide the MLE estimate to the standard fitting processes, restrict the number of iterations to zero and abuse the existing algorithms to get standard error estimates. This solution, however, should not be regarded as a long-term solution to the problem of failed convergence in log-binomial models. There are newly emerging optimization techniques in the field of applied mathematics that may solve this problem altogether [14,20,23]. These methods may be better suited for log-binomial models than the standard Newton-Raphson methods that are currently used. Nevertheless, in the face of failed convergence a brute force approach can be easily taken while the necessary research is done to investigate these other methods for estimating log-binomial models.

## Conclusions

With the increasing attention on estimating relative risk using log-binomial models there will be increasing circumstances where researchers encounter a non-convergent log-binomial model. Granted this paper does not present every possible scenario where failed convergence may occur; however, from the simple examples presented above it seems clear that there are situations where failed convergence may occur despite the model being estimable through less standard or familiar methods. In these circumstances researchers should not simply abandon their decision to use a log-binomial model but should consider a more careful examination of possible causes. For example, one might consider a reparametrization of the model if it known that the MLE resides on a boundary. In another case, a researcher may elect to attempt a brute force search of the parameter space for the MLE. Certainly, further research is needed on the estimation methods for log-binomial models, perhaps borrowing some of the recent development from the applied mathematicians on the subject. However, the message from this paper should be clear, when log-binomial models fail to converge, do not give up.

## Appendix 1 - Statistical software

An investigation of the viability of log-binomial models is inseparably connected with consideration of the current state of statistical software. Indeed this current state is changing rapidly. Nevertheless, we felt it appropriate to provide some cautious comparisons in part to support our view that log-binomial models per se are not the issue, it is the current implementations that are available. Widespread use of the method will not be accomplished until the method is implemented in the standard statistical packages, in a reliable way. The most widely used, and hence influential, statistical packages for health research and many other disciplines are R, STATA, SAS and SPSS. This appendix is an informal exploration of the differences between these packages as it pertains to the estimation of log-binomial models. We recognize that there are many other software systems and not attempted to be inclusive here. Ultimately, none of these software packages are adequate for modelling log-binomial models in their current state to ensure results that can be trusted.

### R

The R statistical package [15] is at the forefront of statistical computing by virtue of being open-source. Usually, R provides maximal control over the estimating algorithms as compared to the proprietary alternatives and includes all the functionality of each of them and more. Further, since it is open-source it is widely available, and thus not prohibitive from an access point of view. For these reasons, R was generally the first choice for the software used in this work.

With respect to the GLM fitting algorithms in R, an advantage to the fitting process that has not been identified in any of the other packages is that for log-binomial models specifically there is a functionality included that ensures that the fitted values are within the allowable space. This is a logical check to ensure that the fitted values are always negative. If positive fitted values are encountered early in the estimation process then the fit is halted and better starting values are requested from the user. No other package offered this. Also, R makes use of a procedure known as step-halving. If a positive fitted value would be produced during the fitting process (i.e., the iteration has tried to move outside the parameter space) the update is halved and the fitted value is recomputed. If the fitted value is still positive the update is halved again and again until the fitted value is negative. Usually this only requires a single step halving. In combination, these methods guarantee that if convergence is reported it must be to a value inside the parameter space.

### STATA

In contrast to R, STATA [16] does not have a check in place to ensure that the process only iterates inside the parameter space. While not shown here, we have observed examples where STATA converges to a place outside the allowable parameter space. This is a consequence of the fact that the numerical optimization is completely unconstrained. The problem with that is that the log-likelihood function is not defined outside the parameter space so computing a ‘log-likelihood’ value for a point outside the parameter space is non-sense. The STATA reference manual [24] alludes to the fact that the numerical methods used to fit log-binomial models are actually based on the method proposed by Wacholder [8]; however, evidence of this has yet to be observed. In fact, when the authors observed failed convergence of models fit with STATA, the failed convergence was a consequence of the iteration going to an illegal place and never returning to the parameter space. More research is needed looking at the log-binomial fitting algorithms in STATA.

One of the advantages of STATA, however, is that both the observed and expected Hessians can be used in the fitting process. This can be done exclusively with one or the other, or through a combination of the two. STATA allows the user to specify the number of IRLS iterations or the number of iterations using the observed Hessian (called *ml* iterations in STATA). This increased flexibility is a bonus; however, there are conditions where either method will fail and with STATA allowing the iterations to wander outside the parameter space this is usually a moot point.

When the estimating software is supplied with starting values that are effectively the MLE, from the brute force maximization, occasionally convergence would be reported in STATA. The other advantage of STATA is that when a model is estimable, STATA provides a number of options for estimating the standard error. The observed or expected information is available as well as jackknife and bootstrap methods and the robust method known as the Huber-White sandwich estimate [25].

### SAS

The options available with estimation in SAS [17] is very similar to those in STATA. SAS does not have a parameter space check like R does and can iterate outside the allowable space. One advantage of SAS over STATA is that after a pre-set number of iterations if the estimation algorithm has not been judged to be any closer to the MLE then the optimization is ceased and the user is notified. In STATA, the iterative process continues without end until the process is killed by the user.

SAS was able to correctly converge to the MLE in the example given in Table 3 of the manuscript. However, we have been able to determine that SAS will still fail to converge in other similar examples and converge to an illegal place (i.e., outside the parameter space) and stop. Like other systems, SAS will report an error indicating that there were illegal fitted values for at least one observation in the dataset but providing invalid model estimates offers approximately the same value as reporting failed convergence.

### SPSS

The statistical package SPSS [18] is similar to all the others already considered. Maximization is done via Newton-Raphson using either the observed or expected Hessian or a combination of iterations using one or the other [26]. Initial values need not be supplied as the estimation procedure will compute initial values for the parameters but initial values can be supplied by the user. The SPSS implementation also makes use of step-halving, similar to R. Other characteristics of the implementation in SPSS are almost identical to the others, convergence tolerances and so forth are generally common and user adjustable as necessary.

However, in spite of a large number of similarities with the other packages, the failure of SPSS in relation to the example given in Table 3 is unique. SPSS, with the default convergence options, proceeds through 6 iterations and then declares convergence to a point outside the parameter space. Fortunately it offers a warning much like the one provided in SAS when landing in an illegal place indicating that there are “invalid cases” in the dataset.

## Appendix 2 - Technical appendix

This section presents the technical specifics of the example provided in Table 3 of the manuscript. It is assumed that the model of interest is a log-binomial model with a single linear predictor *X* which has three possible values, *X* = -1,0,1. The model of interest is

$$\log(p)=\beta_{0}+\beta_{1}X$$

which is labelled as model 3 above. For this model, the log-likelihood function is given as

$$\begin{array}{lll} l(\beta )=18\beta_{0} & +17\log(1-e^{\beta_{0}+\beta_{1}})+3\log(1-e^{\beta_{0}})\; & \;\\ & +2\log(1-e^{\beta_{0}-\beta_{1}})\; & \; \end{array}$$

with the score functions for *β*_0_ and *β*_1_ given respectively by

$$S_{0}=\frac{\mathrm{\partial l}(\beta_{0},\beta_{1})}{\partial \beta_{0}}=18-\frac{17e^{\beta_{0}+\beta_{1}}}{1-e^{\beta_{0}+\beta_{1}}}-\frac{3e^{\beta_{0}}}{1-e^{\beta_{0}}}-\frac{2e^{\beta_{0}-\beta_{1}}}{1-e^{\beta_{s}-\beta_{1}}}$$

and

$$S_{1}=\frac{\mathrm{\partial l}(\beta_{0},\beta_{1})}{\partial \beta_{1}}=\frac{17e^{\beta_{0}+\beta_{1}}}{1-e^{\beta_{0}+\beta_{1}}}+\frac{2e^{\beta_{0}-\beta_{1}}}{1-e^{\beta_{0}-\beta_{1}}}.$$

The observed Hessian is given as

$$H=\left[\begin{array}{ll} \frac{\partial^{2}l(\beta_{0},\beta_{1})}{\partial \beta_{0}^{2}} & \frac{\partial^{2}l(\beta_{0},\beta_{1})}{\partial \beta_{1}\beta_{0}}\\ \frac{\partial^{2}l(\beta_{0},\beta_{1})}{\partial \beta_{0}\beta_{1}} & \frac{\partial^{2}l(\beta_{0},\beta_{1})}{\partial \beta_{1}^{2}} \end{array}\right]=\left[\begin{array}{ll} H_{00} & H_{01}\\ H_{10} & H_{11} \end{array}\right]$$

where

$$H_{00}=\frac{-17e^{\beta_{0}+\beta_{1}}}{{\left(1-e^{\beta_{0}+\beta_{1}}\right)}^{2}}-\frac{3e^{\beta_{0}}}{{\left(1-e^{\beta_{0}}\right)}^{2}}-\frac{2e^{\beta_{0}-\beta_{1}}}{{\left(1-e^{\beta_{0}-\beta_{1}}\right)}^{2}},$$

$$H_{10}=H_{01}=\frac{-17e^{\beta_{0}+\beta_{1}}}{{\left(1-e^{\beta_{0}+\beta_{1}}\right)}^{2}}+\frac{2e^{\beta_{0}-\beta_{1}}}{{\left(1-e^{\beta_{0}-\beta_{1}}\right)}^{2}},$$

and

$$H_{11}=\frac{-17e^{\beta_{0}+\beta_{1}}}{{\left(1-e^{\beta_{0}+\beta_{1}}\right)}^{2}}-\frac{2e^{\beta_{0}-\beta_{1}}}{{\left(1-e^{\beta_{0}-\beta_{1}}\right)}^{2}}.$$

## Abbreviations

RR: Relative risk; OR: Odds ratio; MLE: Maximum likelihood estimate; GLM: Generalized linear model.

## Competing interests

The authors declare that they have no competing interests.

## Authors’ contributions

TW carried out the simulations, analysed the data, and drafted the manuscript. ME initially proposed the idea and contributed to the study design. GHF contributed to the simulation studies and the study design and assisted in drafting the manuscript. All authors read and approved the final manuscript.
